# Macroevolutionary constraints on global microbial diversity

**DOI:** 10.1002/ece3.10403

**Published:** 2023-08-08

**Authors:** Ford J. Fishman, Jay T. Lennon

**Affiliations:** ^1^ Department of Biology Indiana University Bloomington Indiana USA

**Keywords:** bacteria, diversification, macroecology, mass extinction, microbiome, speciation, species richness

## Abstract

Biologists have long sought to quantify the number of species on Earth. Often missing from these efforts is the contribution of microorganisms, the smallest but most abundant form of life on the planet. Despite recent large‐scale sampling efforts, estimates of global microbial diversity span many orders of magnitude. It is important to consider how speciation and extinction over the last 4 billion years constrain inventories of biodiversity. We parameterized macroevolutionary models based on birth–death processes that assume constant and universal speciation and extinction rates. The models reveal that richness beyond 10^12^ species is feasible and in agreement with empirical predictions. Additional simulations suggest that mass extinction events do not place hard limits on modern‐day microbial diversity. Together, our study provides independent support for a massive global‐scale microbiome while shedding light on the upper limits of life on Earth.

## Graphical abstract

Microorganisms have traditionally been overlooked when estimating the number of species on Earth. We parameterized macroevolutionary models, which indicate that global richness beyond one trillion (10^12^) species is feasible and in agreement with empirical predictions.

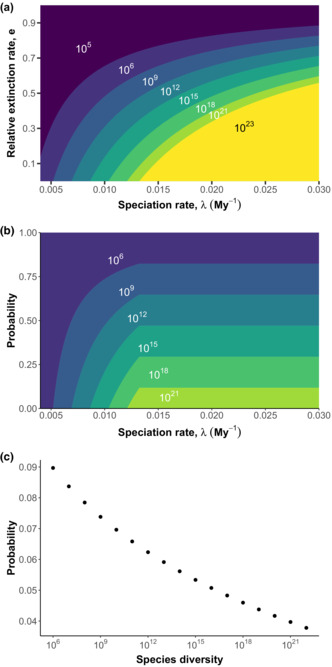

## 1 INTRODUCTION

For many decades, ecologists and evolutionary biologists have attempted to predict the number of species on Earth (May, 1988). Such estimates can be useful for conservation and biodiversity efforts, while also shedding light on the dynamics and balance of speciation and extinction on a planetary scale (May, 2011). Global biodiversity is impossible to completely census, given the large number of individuals across a diverse range of habitats (Whitman et al., 1998). Various approaches have been used instead to approximate biodiversity, including diversity estimation based on partial censuses (Costello et al., 2012; Louca et al., 2019), ratios of taxonomic groupings (Mora et al., 2011), and many other macroecological and biogeographical methods (May, 1988). The total number of species on the planet, when focusing on multicellular life, has been estimated to range between 10^6^ and 10^9^ species (Costello et al., 2012; Li & Wiens, 2022; Mora et al., 2011; Thompson et al., 2017). Despite their abundance and ubiquity, microorganisms have historically been overlooked when attempting to estimate global biodiversity (Larsen et al., 2017). This oversight was largely due to technological limitations, as there were no comprehensive methods to systematically describe microbial diversity (Woese, 1987).

With the advent of high‐throughput DNA amplicon sequencing, large‐scale quantification of microbial diversity became possible, yet the various approaches used to predict the total number of microbial species have generated highly divergent estimates (Larsen et al., 2017; Locey & Lennon, 2016; Louca et al., 2019). Bacteria and archaea can be clustered into operational taxonomic units (OTUs) according to their similarity in the 16S rRNA gene, with the cutoff often being set to 97% (Stackebrandt & Goebel, 1994). While this clustering approach is often considered a conservative measure (Eren et al., 2015; Poretsky et al., 2014), the 16S rRNA has served as a powerful and widely used tool, allowing microbiologists to survey the diversity of bacteria and archaea in a range of ecosystems across the planet (Thompson et al., 2017). Using the massive amount of data collected, several studies have attempted to quantify global bacterial and archaeal species richness (*S*). One approach using collector's curves estimated that microbes may add 10^6^ species to the inventory plant and animal diversity (Louca et al., 2019), which is on the same order of magnitude as fungal diversity (Hawksworth & Lucking, 2017). If accurate, this would mean that the inclusion of microbial life would not fundamentally change current estimates of global macroorganismal diversity, which tend not to exceed 10^7^ (Mora et al., 2011). Another approach using the average number of unique bacterial species per host species estimated global *S* to be approximately 10^9^ (Larsen et al., 2017; Li & Wiens, 2022; Wiens, 2021). Last, a combination of scaling laws and biodiversity theory predicts that there are 10^12^ or more microbial taxa on Earth (Lennon & Locey, 2020; Locey & Lennon, 2016), including potentially 10^9^ in activated sludge systems alone (Wu et al., 2019). This ongoing debate is unresolved, as these predictions and estimates are difficult to directly test, but it may be possible to deem some values of present‐day diversity as impossible given our current understanding of biodiversity.

There are a number of potentially important factors that could constrain global microbial diversity. The abundance of microorganisms (*N*) at a global scale has approached a steady state of 10^29^–10^30^ individuals (Kallmeyer et al., 2012; Whitman et al., 1998). Given that global taxon richness *S* cannot exceed the number of total individuals *N*, *S*_present_ ≤ 10^30^ is a hard upper constraint on microbial richness. However, there may also be a soft upper constraint of 10^22^–10^23^ due to neutral drift, if one assumes a constant 10^30^ prokaryotic (bacteria and archaea) individuals and a neutral drift rate of 4–5 × 10^−9^ substitutions per site per generation (Louca et al., 2019). A hard lower constraint of *S*_present_ ≥ 10^6^ is also in place due to the number of reported 97% 16S rRNA OTUs (Schloss et al., 2016). Therefore, it is reasonable to surmise that the present number of bacterial and archaeal taxa *S*_present_ is between 10^6^ and 10^23^.

Within this range, diversity is further constrained by macroevolutionary processes occurring over geological timescales. Speciation and extinction rates of lineages, the difference of which is the net diversification rate, should directly influence total present microbial diversity (Scholl & Wiens, 2016) and determine the feasibility of both high and low estimates of this diversity. The simplest diversification models are birth–death processes, which assume constant and universal speciation and extinction rates (Raup, 1985), but more complicated models should address realistic variation in these rates, such as clade‐specific diversification rates (Moran et al., 1995; Scholl & Wiens, 2016). Among macroorganisms, well‐documented mass extinction events are another way the assumption of constant diversification is not upheld (Raup & Sepkoski, 1982; Rohde & Muller, 2005). These include the “Big Five” mass extinction events that eliminated 50%–90% of marine invertebrate genera (Raup & Sepkoski, 1982), as well as the Great Oxidation Event (GOE; Gumsley et al., 2017), which likely caused the mass extinction of many lineages as Earth's atmosphere was transformed over a period of 400 million years owing to the evolution of oxygenic photosynthesis (Hodgskiss et al., 2019). Each of these mass extinction events may have reduced microbial diversity, thus constraining contemporary microbial richness, as a large portion of bacterial diversity is likely host‐associated (Hernández‐Hernández et al., 2021; Thompson et al., 2017; Xie et al., 2005). The same factors causing the mass extinction of macroorganisms may also have elevated free‐living microbial extinction (Newby et al., 2021), though it is reasonable to assume that the diversity of host‐associated taxa should have been most greatly reduced. Models accounting for these phenomena may minimize uncertainty about the number of modern‐day microbial taxa, and also address questions pertaining to the upper limits of global diversity.

To understand how macroevolutionary rates constrain species diversity today, unbiased estimates of speciation and extinction are necessary. Any existing estimates of microbial diversification are derived from phylogenetic data (Louca et al., 2018; Scholl & Wiens, 2016). Due to the nearly nonexistent microbial fossil record, these phylogenies are constructed solely from molecular data, which may lead to incorrect rate estimation when diversification rates vary among lineages (Rabosky, 2010; Stadler, 2009). These phylogenies can also be generated by highly dissimilar birth–death processes that have divergent speciation and extinction dynamics (Louca & Pennell, 2020). Such methods also require estimates for total microbial richness and the number of unsampled taxa to calculate diversification rates (Louca et al., 2018), which would run counter to the aim of using diversification rates to constrain present‐day microbial richness. Therefore, diversification rate estimates that do not estimate unsampled taxa and that are not derived from molecular data alone are necessary to understand macroevolutionary constraints on species richness (*S*).

In this study, we seek to understand how speciation and extinction rates put additional constraints on present‐day microbial diversity. To do so, we estimated speciation rates without phylogenetic inference to avoid the biases discussed above. With a simple model of diversification, we show the probability of various levels of present‐day diversity. We then modify this model to account for mass extinction events to explore their potential effects on global diversity. Our findings are potentially valuable because they provide an independent means for evaluating the feasibility of some empirical estimates of global biodiversity.

## 2 METHODS

### 2.1 Rate estimation

In order to explore bacterial and archaeal richness, we must first consider our species definition. We phylogenetically defined a species as a cluster of strains with 97% 16S rRNA sequence similarity. While the 16S rRNA has limitations differentiating between certain taxa (Poretsky et al., 2014), its broad conservation across bacteria and archaea, along with its relatively slow rate of evolution, makes it a convenient biomarker for considering global bacterial and archaeal diversity (Woese, 1987). Over evolutionary time, the accumulation of 16S rRNA substitutions can cause a focal sequence and an ancestral sequence to diverge by at least 3%. This divergence can be considered a proxy for a speciation event. Thus, speciation rate *λ* can be calculated using 16S rRNA nucleotide substitution rates (*K*_16S_) as follows:

(1) $$\lambda =\frac{16S\;\text{length}\times K_{16S}}{3\%\times 16S\;\text{length}}.$$

In Equation (1), the numerator represents the total number of substitutions a 16S sequence undergoes over a million years, and the denominator represents the total number substitutions necessary for a 3% divergence in sequence, which is a speciation event according to an OTU species definition. The bacterial taxa used to calculate these substitution rates belong to the Gammaproteobacteria (*Buchnera*, *Carsonella*, *Portiera*, *Wigglesworthia*) and Flavobacteria (*Blattabacterium* and *Sulcia*) (Kuo & Ochman, 2009).

To calculate *λ* values, we used a range of *K*_16S_ values (0.025%–0.091% divergence/nt/My) based on the divergence of endosymbiotic bacteria in preserved and dated insects (Kuo & Ochman, 2009). These *K*_16S_ values were calculated by calibrating the bacterial phylogenies with the age of their insect hosts, which possess a tractable fossil record (Moran et al., 1993). In this way, the ages of internal nodes of the bacterial phylogeny were mapped to corresponding ages in the insect phylogeny. These ages and the divergence between two bacterial 16S rRNA sequences were then used to directly calculate *K*_16S_. Using these *K*_16S_ values, we calculated speciation rates of 0.0083–0.030 My^−1^. However, because substitution rates of endosymbiont bacteria are potentially twice that of their free‐living relatives (Moran et al., 1995), we also considered speciation rates 50% smaller than the minimum endosymbiont‐based speciation rate, producing a final range of 0.004–0.03 My^−1^. As an analogous technique cannot be used to estimate extinction rates (*μ*), we used values of relative extinction rates *ε*, the ratio of extinction to speciation (*μ*/*λ*), between 0 and 1 to account for various extinction scenarios.

### 2.2 Expectations of birth–death process

The process of lineage diversification is often modeled as a stochastic birth–death process (Magallón & Sanderson, 2001; Nee et al., 1994; Raup, 1985), where speciation and extinction events are analogous to births and deaths of individuals, respectively. In diversification scenarios where present‐day *S ≥* 10^12^ taxa, the simulation of a stochastic birth–death process that stores times of birth and death events becomes computationally intractable. Due to this limitation, we first analyzed the expectations of birth–death processes *E*[*S*_*t*_] with constant speciation and extinction rates, which can be simply described by exponential growth when assuming the initial number of species to be 1:

(2) $$E\left[S_{t}\right]=e^{\left(\lambda -\mu \right)t}.$$

To compare the amount of diversity across various levels of *λ* and *ε*, we manipulated Equation (2) into the following:

(3) $$\varepsilon \left(\lambda \right)=1-\frac{\ln\left(E\left[S_{t}\right]\right)}{\mathrm{\lambda t}}.$$

We plotted several contours of *ε*(*λ*) with various levels of *E*[*S*_*t*_] with *t* = 4000 My, a reasonable estimate of the time passed since the last universal common ancestor (Weiss et al., 2018). To calculate the probability of various ranges of present‐day diversity, we calculated the area between contours via integration and normalized by the total area of feasible parameter space (10^6^ ≤ *E*[*S*_*t*_] < 10^23^). See Table 1 for parameters and descriptions used in this birth–death process model.

**TABLE 1:** Defining key model parameters used in birth–death process and mass extinction models.

Variable/parameter	Description
Global species diversity (*S*_*t*_)	The total number of 97% 16S rRNA bacterial and archaeal operational taxonomic units (i.e., richness) present on the planet at time *t*
Speciation rate (*λ*)	The number of species an extant species generates per million years (My^−1^)
Extinction rate (*μ*)	Number of species extinctions per extant species per million years (My^−1^)
Relative extinction rate (*ε*)	Ratio of extinction rate to speciation rate (*μ*/*λ*)
Mass extinction intensity (*p*)	The proportion of vulnerable species removed at a certain timestep from a mass extinction event
Vulnerable proportion of taxa (*q*)	The proportion of species that are vulnerable to mass extinction
Mass extinction events (*M*)	The set of timesteps where mass extinction occurs
Host‐associated mass extinction events (*M*_H_)	The set of timesteps where mass extinction of hosts occurs

### 2.3 Mass extinction events

To understand the potential influence of mass extinction and its ability to constrain present‐day microbial diversity, we considered the following mass extinction events: the Great Oxidation Event (GOE; ~2450 Mya) (Gumsley et al., 2017), the Ordovician‐Silurian (O‐S, 445 Mya), the Devonian (D, 375 Mya), the Permian–Triassic (P‐Tr, 252 Mya), the Triassic‐Jurassic (Tr‐J, 201 Mya), and the Cretaceous (K‐T, 66 Mya) (Gumsley et al., 2017; Raup & Sepkoski, 1982). Our model of mass extinction uses the expression for the expectations of a birth–death process (Equation 2) and adds additional terms accounting for mass extinction events. Specifically, we consider two new parameters influencing extinction beyond constant extinction rate *μ*: the intensity of mass extinction (*p*) and the proportion of taxa potentially affected by mass extinction (*q*). We make this distinction to model a situation where only host‐associated taxa are vulnerable to mass extinction, as these mass extinction events correspond to host extinction (*q* < 1). This way, the effect of mass extinction can be modeled separately from the effect of host‐associated taxa. For each mass extinction event, there is a single reduction in the total number of species according to the magnitudes of *p* and *q*. To obtain the number of species at time *t*, we multiply the birth–death expectations (Equation 2) by the proportion of taxa surviving each mass extinction event (1 − *pq*) for as many mass extinction events occurring by time *t*, where *t* is some nonnegative integer:

(4) $$S_{t}=e^{\left(\lambda -\mu \right)t}\prod_{i=1}^{t}1-p\left(i\right)q\left(i\right).$$

Let *M* be the set of the six timesteps where mass extinction occurred, and *M*_H_ be the set of timesteps with host‐associated mass extinction. Mass extinction intensity *p*(*i*) is equal to some value *p* during a mass extinction event and 0 for all other timesteps:

(5) $$p\left(i\right)=\{p\;\text{if}\;i\in M\;0\;\text{otherwise}.$$

We consider situations where *p* is 0.0, 0.5, or 0.9 to model situations without mass extinction, with moderate mass extinction, and with intense mass extinction, respectively. Likewise, the proportion of taxa vulnerable to mass extinction *q*(*i*) at timestep *i* is set to some value *q* during a host‐associated mass extinction event and 1 for all other timesteps:

(6) $$q\left(i\right)=\{q\;\text{if}\;i\in M_{H}\;1\;\text{otherwise}.$$

Therefore, the proportion of species removed due to mass extinction is *pq* during host‐associated mass extinction, *p* during nonhost‐associated mass extinction, and 0 for all other timesteps. When considering Equations (5) and (6) when *t* = 4000 My (present day), the Great Oxidization Event is the only nonhost‐associated mass extinction event, so there is one timestep where the proportion of species removed is *p* and five timesteps where the proportion is *pq*. Equation (4) then becomes

(7) $$S_{4000}=e^{\left(\lambda -\mu \right)\times 4000}\left(1-p\right){\left(1-\mathrm{pq}\right)}^{5},$$

which we further transformed to produce contours of *ε* in terms of *λ*:

(8) $$\varepsilon \left(\lambda \right)=1-\frac{\ln\left(S_{4000}\times {\left(1-p\right)}^{-1}{\left(1-\mathrm{pq}\right)}^{-5}\right)}{4000\lambda }.$$

To obtain informed estimates of *q*, we assumed that host‐associated bacteria and archaea would be more likely to go extinct from macroorganismal mass extinction than would free‐living microbes. In place of the unmeasurable ancient proportion of host‐associated species, we calculated the present‐day proportion of host‐associated microbial species, as well as obligately host‐associated and preferentially host‐associated proportions, using observation tables for 90 base pair OTUs and sample metadata from the Earth Microbiome Project (EMP; Thompson et al., 2017). All samples labeled in the metadata as “Free‐living” were not taken from a host, and all others were taken from hosts. We defined obligately host‐associated taxa as OTUs only sampled from hosts and preferentially host‐associated taxa as OTUs found in hosts for over 50% of their total occurrences. See Table 1 for parameters and descriptions used for mass extinction modeling.

## 3 RESULTS

### 3.1 Expectations of birth–death process

**FIGURE 1:**
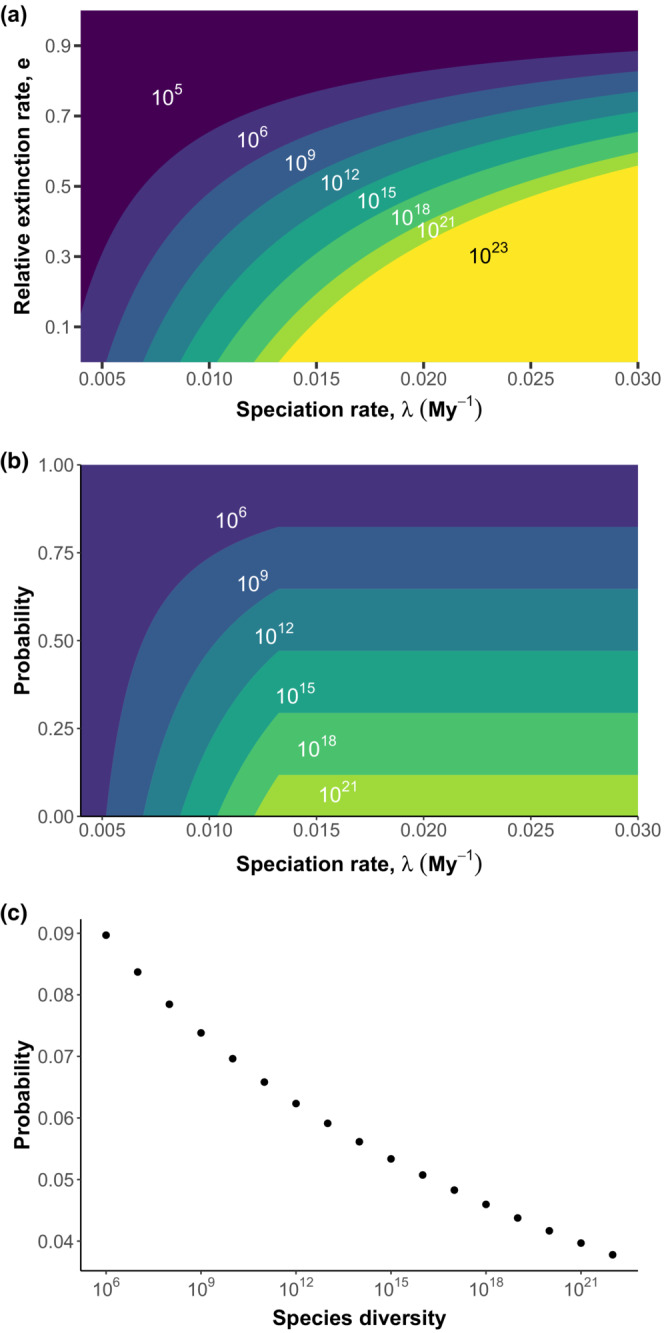
Expected present‐day number of species (*E*[*S*_4000_]) generated from a birth–death process with probabilities of certain outcomes. (a) Combinations of speciation (0.004 ≤ *λ* ≤ 0.03) and relative extinction rates (0 ≤ *ε* ≤ 1) lead to a wide range of *E*[*S*_4000_]. The regions labeled 10^5^ and 10^23^ consist of combinations of diversification parameters that lead to infeasibly low (*S* < 10^6^) and infeasibly high (*S* > 10^23^) species diversity, respectively. All other labeled regions correspond to *E*[*S*_4000_] within a three order of magnitude bin (e.g., 10^6^ label corresponds to 10^6^–10^9^ species) except 10^21^ (10^21^–10^23^ species). Each labeled region is separated by contours of *ε*(*λ*) (Equation 3) with values of *E*[*S*_4000_] between 10^6^ and 10^23^ species. (b) The probability of diversity outcome bins across speciation rate. The probability is calculated as the proportion of *ε* values leading to a diversity outcome (e.g., 10^6^–10^9^ species) at a given value of *λ*. (c) The overall probability of diversity outcomes spanning one order of magnitude. This probability was calculated by creating contours of *ε*(*λ*) (Equation 3) with *E*[*S*_4000_] set from 10^6^ to 10^23^ and calculating the area between each contour and normalizing by the total area of the feasible parameter space.

To evaluate how diversification parameters constrain present‐day microbial species richness, we expressed relative extinction rate (*ε*) as a function of speciation rate (*λ*) at various contours of *E*[*S*_*t*_] (Equation 3) and with *t* = 4000 My (present‐day) within the bounds of the speciation rate range described above (Figure 1). Expected diversity increases as extinction decreases and speciation rises, though the relationship between *ε* and *λ* is nonlinear (Equation 5). Approximately 50% of the combinations of *λ* and *ε* lead to infeasibly low (<10^6^) or high (>10^23^) diversity (Table S1). Certain high levels of diversity require *ε* to be sufficiently low and *λ* to be sufficiently large (Figure 1a,b). For instance, 10^12^ species are only possible for *ε* < 0.78 and *λ* > 0.007 sp. My^−1^. However, limitations on *ε* or *λ* for 10^6^ species are much less strict. In terms of feasible parameter space, lower diversity outcomes are somewhat more likely than high diversity outcomes (Figure 1c). For instance, the probability of 10^6^–10^7^ species is ~9.0%, while the probability of 10^12^–10^13^ species is ~6.2%. A reason for the decrease in probability for higher levels of diversity is that it is impossible to reach them at relatively low values of *λ* (Figure 1b). However, once *λ* reaches ~0.013 sp. My^−1^, any further increase in *λ* does not alter the relative probabilities of each outcome (Figure 1b). However, each of these ranges of diversity is well within these constraints and far from extreme outcomes, and no outcome is far more probable than the others. This analysis demonstrates that vast diversity is indeed possible within the specified speciation constraints.

### 3.2 Mass extinction events

**FIGURE 2:**
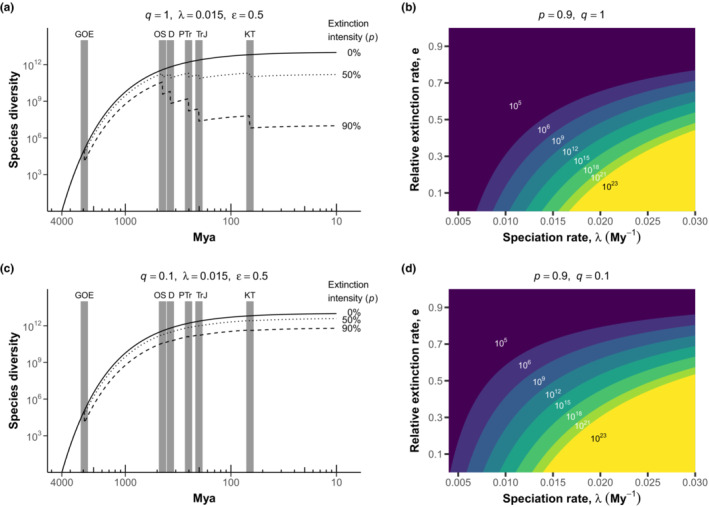
Effect of mass extinction events on species diversity (a, b) when all taxa are vulnerable to mass extinction (*q* = 1) and (c, d) when only obligate host‐associated taxa are vulnerable (*q =* 0.1). (a, c) Species richness over time was calculated using the mass extinction model (Equation 4) with speciation and relative extinction at *λ* = 0.015 My^−1^ and *ε* = 0.5, respectively. Known mass extinction events (gray bars) cause a one‐time 0% (solid), 50% (dotted), or 90% (dashed) reduction in diversity of vulnerable species at each mass extinction event (D, Devonian; GOE, Great Oxidation Event; KT, Cretaceous; OS, Ordovician‐Silurian; PTr, Permian–Triassic; TrJ, Triassic‐Jurassic). (b, d) Present‐day diversity calculated from mass extinction model across a range of speciation (0.004 ≤ *λ* ≤ 0.03) and relative extinction rates (0 ≤ *ε* ≤ 1) with mass extinction intensity *p* = 0.9. Each labeled region (same coloration scheme as Figure 1a) is separated by contours of *ε*(*λ*) (Equation 8) with values of *E*[*S*_4000_] between 10^6^ and 10^23^.

Our simulations show that the intensity of mass extinction events determines their effect on present microbial biodiversity (Figure 2). Let us first consider scenarios where mass extinction affects all species equally (*q* = 1) and strongly (*p* = 0.9; Figure 2a,b). Compared to scenarios without mass extinction, much higher speciation rates and lower extinction rates are required to reach equivalent levels of richness (Figures 1a and 2b). For example, net diversification parameters resulting in 10^12^ species when *p* = 0 lead to ~10^6^ species when *p* = 0.9 (Figure 2a). Additionally, the proportion of total feasible parameter space decreases from 50.6% in the birth–death expectation model to 36.5% in the mass extinction model (Table S1). The proportion of parameter space leading to >10^6^ species increases to 50.2% from 26.7%, as well (Table S1). However, the relative probabilities of each diversity outcome within the feasible parameter space are primarily unchanged (Figure S1). Setting *p* = 0.9 is comparable to the degree of extinction in macroorganisms during the Permian–Triassic, the most severe mass extinction event (Sepkoski, 1990). If these events lead to even a 50% diversity reduction, present‐day diversity still is decreased, though the effect is diminished compared to the 90% scenario. However, it is clear that severe mass extinction can greatly change the outcomes for individual parameter combinations.

To obtain an informed estimate of the proportion of taxa vulnerable to mass extinction (*q*), we used species and site data from the EMP to calculate the present‐day proportion of host‐associated 16S OTUs. We found that ~90% of EMP bacterial OTUs were free‐living to some degree, meaning that only ~10% of EMP OTUs were obligately host‐associated (Table 2). However, about half of all OTUs were host‐associated to some degree. Therefore, if mass extinction events of macroorganisms only resulted in the extinction of host‐associated organisms, only between 10% and 50% of microbial taxa would be vulnerable to mass extinction, depending on if all host‐associated species, only obligately host‐associated species, or some mixture is considered.

**TABLE 2:** Proportion of Earth Microbiome Project (EMP) 16S rRNA operational taxonomic units (OTUs) classified as host‐associated or free‐living.

Niche	Proportion of all EMP OTUs (%)
Host‐associated	47.8
Preferentially host‐associated	19.9
Obligate host‐associated	9.3
Free‐living	90.7
Preferentially free‐living	78.9
Obligate free‐living	52.2

*Note*: Obligate host‐associated OTUs are only found in samples taken from hosts, as opposed to preferentially host‐associated (over 50% of samples from hosts) and host‐associated OTUs (any samples are from hosts). The same terms are applied to free‐living OTUs.

When we consider scenarios when only a fraction of microbial species is affected by mass extinction, the model begins to converge to the expectations of the birth–death processes (Figure 2c,d). With *q* = 0.1, which corresponds to only 10% of microbial lineages being vulnerable to mass extinction, mass extinction has a much more muted effect. The same net diversification parameters described above resulting in 10^12^ species when *p* = 0 now leads to >10^10^ species at *p* = 0.9. The feasible parameter space also converges to that of the birth–death expectations as well (Table S1, Figures 1a and 2d). We also modeled scenarios where only certain groupings of lineages were vulnerable to mass extinction and identified scenarios where this allows for present‐day diversity on the order of the birth–death expectations (Figure S2), illustrating another scenario where adding biological detail can reduce the effect of mass extinction. Therefore, while extreme mass extinction scenarios may constrain microbial diversity, more conservative scenarios suggest that mass extinction may have only moderately decreased present‐day richness.

## 4 DISCUSSION

In this study, we modeled how macroevolutionary rates influence present‐day microbial species diversity in an attempt to further constrain the estimates and predictions from previous studies ranging from 10^6^ to 10^12^ species (Larsen et al., 2017; Locey & Lennon, 2016; Louca et al., 2019; Wiens, 2021) to see whether some of these estimates are macroevolutionarily infeasible. The values suggested in these studies all can be generated from feasible combinations of macroevolutionary rates (Figure 1). In fact, our results introduce the possibility that bacterial and archaeal diversity may outstrip the largest predictions (Lennon & Locey, 2020; Locey & Lennon, 2016). Given the diversification parameters and the model we used, 10^6^ species is slightly more likely than 10^9^, both of which are more likely than 10^12^. However, while our study finds that it is most likely that total present‐day diversity is not orders of magnitude larger than current inventories, it does not deem any previously made prediction or estimate vanishingly unlikely or even improbable (Larsen et al., 2017; Lennon & Locey, 2020; Locey & Lennon, 2016; Louca et al., 2019; Wiens, 2021). Importantly, we do not suggest that microbial diversity is limitless. Rather, we emphasize that these models do not suggest that microbial diversity is necessarily limited to the number of OTUs currently described.

The simple approach we use in this study is not without its caveats and assumptions. This study only uses substitution rate data from obligately host‐associated taxa, which may not be representative of the overall rate of molecular evolution of free‐living microbes (Espejo & Plaza, 2018; Moran et al., 1995). While a more representative sample of substitution rates from free‐living lineages or taxa with multiple 16S rRNA copies may improve upon this study, such data come without the time‐calibration of host lineages. Thus, our analysis provides a basis for feasible levels of microbial diversity with backing from the fossil record and including speciation values that account for the differences between free‐living and obligate endosymbiont substitution rates (Moran et al., 1995). Additionally, we did not attempt to directly estimate extinction rates, as microbial extinction cannot reasonably be estimated apart from phylogenetic approaches relying on a priori assumptions of microbial richness (Louca et al., 2018), which given the objectives of our study would introduce circular reasoning. If an unbiased method for estimating relative extinction was identified, then it could be used to further constrain the diversification parameter space. Our analysis also assumes no biogeographical or niche association with diversification (Li & Wiens, 2022). It is quite likely that microbial diversification rates vary greatly across different clades, as has been described in plant and animal systems (Rabosky, 2020). While clade‐specific diversification rates were modeled here, a more thorough modeling process including diversification dynamics of specific bacterial lineages may provide more insight into global diversification.

Our simulations of mass extinction events showed that while severe mass extinction can constrain present‐day diversity, there are many scenarios that result in little change compared with our model without mass extinction. This convergence to birth–death expectations occurs as the proportion of lineages affected by mass extinction decreases. In fact, the true proportion of bacteria affected by host mass extinction may have been smaller than the proportion of obligately host‐associated taxa depending on the host range of the microbial lineages. For instance, if one microbial taxon is present in several host taxa, extinction is unlikely if only one host taxon becomes extinct. However, the publicly available 16S rRNA databases do not typically contain information regarding whether OTUs were found in a narrow or broad range of host taxa, only the general source of each sample. It is also possible for plant and animal mass extinction to affect more than just host‐associated microbes if higher‐order effects of extinction of macroorganisms had downstream effects on free‐living microbes, thus increasing the possible percentage of microbial taxa vulnerable to mass extinction. However, explicitly modeling such effects here is unnecessary, as the outcomes with high *q* will simply converge to our first mass extinction scenario with *q* = 1.0.

Our mass extinction model contains other assumptions and caveats as well. To simplify the model, we implemented mass extinction as a one‐time reduction in diversity per event. These events might be more realistically modeled as occurring over the span of several million years. We implemented each of the “Big Five” mass extinction events as equal in extinction magnitude, but some of these events had larger effects on host diversity than others (Raup & Sepkoski, 1982), which likely would have scaled onto microbial extinction. However, it is not clear to what degree microbial extinction would increase or decrease with the extinction of macroorganisms. Additionally, there may have been other microbe‐specific mass extinction events besides the GOE that could have had a profound impact on diversity. Our models also do not take into consideration increases in diversification via adaptive radiation following mass extinction (Stroud & Losos, 2016). Despite these caveats, our models provide a foundation for how losing large proportions of diversity several times may have altered present‐day diversity by examining extreme scenarios.

It has previously been shown that simple macroevolutionary models, like the ones used in our study, can greatly overestimate present‐day diversity by several orders of magnitude, even using taxa with well‐parameterized time calibration (Rabosky & Benson, 2021). This limitation is quite pronounced at large evolutionary timescales. While we acknowledge that these are indeed simple models. We do not use them to claim that global richness is orders of magnitude greater than the largest estimates of diversity. Given the data used and the evolutionary time lines modeled here, there is much room for error and inflation in estimating present‐day richness. Despite this inflation, we can see that 10^12^ species is an easily attainable level of richness, rather than being a fringe possibility.

Our study finds vast diversity beyond 10^12^ species is indeed possible and only marginally less likely than lower levels of diversity. While this analysis suggests the globe is most likely to contain fewer than 10^8^ microbial species, our approach cannot make a precise prediction on microbial diversity, nor can it rule out the predictions and estimates made by previous studies (Larsen et al., 2017; Lennon & Locey, 2020; Locey & Lennon, 2016; Louca et al., 2019; Wiens, 2021). The simple models described here use speciation rates calculated from endosymbiotic bacterial substitution rates, which do not have the inherent bias of requiring estimates of unsampled taxa. These models provide a novel angle with which to address the question of global microbial diversity. New approaches will be necessary to confront the lack of consensus in the field as we seek to reconcile the estimations and results put forth, such as methods going beyond 16S rRNA‐based species definitions and embracing the ecological and functional differences among microorganisms (Arevalo et al., 2019). Such approaches may reveal levels of diversity greater than currently estimated.

## AUTHOR CONTRIBUTIONS

**Ford J. Fishman:** Conceptualization (supporting); data curation (equal); formal analysis (lead); methodology (lead); visualization (equal); writing – original draft (lead); writing – review and editing (equal). **Jay T. Lennon:** Conceptualization (lead); data curation (equal); formal analysis (supporting); funding acquisition (lead); investigation (lead); methodology (supporting); project administration (lead); visualization (equal); writing – original draft (supporting); writing – review and editing (equal).

## FUNDING INFORMATION

Research was supported by the National Science Foundation (DEB‐1934554, DBI‐2022049), US ArmyResearch Office Grant (W911NF‐14‐1‐0411, W911NF‐22‐1‐0014, W911NF‐22‐S‐0008), and the National Aeronautics and Space Administration (80NSSC20K0618).

## Supporting information


Appendix S1

## Data Availability

Data and code are available on GitHub (https://github.com/LennonLab/globalmacroevo) and Zenodo (https://doi.org/10.5281/zenodo.8181498).
